# Effects of Ionizing Radiation on Nutrients in Foods

**DOI:** 10.6028/jres.093.078

**Published:** 1988-06-01

**Authors:** Donald W. Thayer, Jay B. Fox, Ronald K. Jenkins, Stanley A. Ackerman, John G. Phillips

**Affiliations:** Eastern Regional Research Center, USDA, ARS, 600 East Mermaid Lane, Philadelphia, PA 19118

Within the dose ranges and conditions envisioned for food irradiation processing, there is little, if any, measurable effect on nutritional value of proteins, amino acids, carbohydrates, and lipids. The protein efficiency ratios of a number of foods radiated at doses adequate to achieve commercial sterility were not significantly altered by the treatments. Though ionizing radiation does produce changes in carbohydrates, lipids, amino acids, and proteins, these reactions are minimized in foods irradiated under proper conditions [1]. It is important to stress that nutrients are protected by their molecular environment from ionizing radiation. These same nutrients in aqueous solution may undergo severe degradation when exposed to doses of ionizing radiation equivalent to those used for the treatment of foods. Irradiation at subfreezing temperatures in the absence of oxygen results in better products and greater retention of vitamins than does irradiation at ambient temperatures in the presence of air. The effects of high dose irradiation of meats *in vacuo* at temperatures of −30 °C or lower on their vitamin content have been reported in many studies.

The reactions of vitamins to ionizing radiation in fresh meats and poultry products irradiated at normal processing temperatures and in the presence of air for increased shelf life and/or elimination of food-borne pathogens are much less well known. This prompted the Food Safety Inspection Service to request the Agricultural Research Service to conduct a study of the effects of ionizing radiation on five vitamins in fresh poultry and pork meats. An experimental design was developed to determine the effects of gamma irradiation in the presence of air at processing temperatures between −20 °C and +20 °C and at doses between 0 and 7.0 kGy on the vitamins cyanocobolamin (B_12_), pyridoxine (B_6_), niacin, riboflavin, and thiamin (B_1_) using response surface methodology [2] to predict the effects with reasonable accuracy in fresh poultry and pork chops. A response surface statistical design was chosen to provide a series of equations allowing the prediction of the effects of radiation treatments on vitamins over the entire area covered by the experimental design. Implicit in the design described below is the assumption that the vitamin content of the samples would not be altered by either freezing nor warming to temperatures of −20 °C or +20 °C, respectively. A radiation dose of 0.5 kGy was included in the study with pork chops because that would be representative for pork irradiated for the inactivation of trichina. The range of radiation doses and the irradiation temperatures were selected to include those which would have the greatest probability of being selected for elimination of food-borne pathogens other than *Clostridium botulinum* as well as for shelf-life extension for the product. Seven fresh center cut Join pork chops (1/2 inch thick) were used for each replicate sample with three replicates for each treatment (radiation dose and uncooked versus pork chops cooked after irradiation). Five replicate samples were used at the zero radiation dose (control) and at 3.5 kGy and 0 °C. Samples were irradiated at temperatures of −20°, −10°, 0°, + 10°, and +20 °C and at doses of 0, 0.5, 1.75, 3.50, 5.26, and 7.0 kGy. The irradiation protocol is illustrated in table 1. The number of replicates indicated is twice that shown above because one-half were analyzed raw and the other after cooking. The protocol followed for poultry was similar except that four breasts per replicate were used and the 0.50 kGy radiation dose was eliminated. The discussion which follows will be limited to a partial presentation of the effects of thiamin as they relate to validity of the experimental design.

The data obtained from the analyses of the samples described above were used to develop response surface equations which would predict the effect of the irradiation dose and temperature on each vitamin over the entire area covered by the design. From the equation for the response surface for thiamin loss the predicted losses in pork chops irradiated at 0 °C and then cooked were as follows: 0 kGy, −1.5% (−0.04%); 0.50 kGy, −10.1% (14.3%); 3.5 kGy, −48.7% (54.3%); and 7.0 kGy, −65.9% (69.7%). The values given in parentheses represent the average of the actual observed values. There was no loss of thiamin in the control on a sample weight basis upon cooking, but there was an overall weight loss of about 30%. The effect of temperature on the degradation of thiamin can be illustrated by comparison of the predicted results obtained at a dose of 3.5 kGy, losses of 32.7 (34.9%) and 63.3% (60.2%) at −20° and +20 °C respectively. The fit of the predicted values to the measured values is indicated by a *R*^2^ value of 0.90 for the equation for the response surface.

The effect of radiation on thiamin was different in poultry from that observed in pork. The response surface equation for thiamin loss in chicken breasts cooked after irradiation predicted the following losses of thiamin at 0 °C: 0 kGy, + 3.2%; 1.0 kGy, −0.38%; 2.0 kGy, −2.2%; 3.0 kGy, −5.9%; 3.5 kGy, −7.4%; and 7.0 kGy, −34.9%. Thus, in the range of greatest interest for the control of salmonella contamination (3.0 kGy) the loss of thiamin was very low especially when compared to losses in pork chops irradiated and cooked in the same manner.

## Figures and Tables

**Table 1:** Pork irradiation experimental design

Temperature	Radiation dose (kGy)					
°C	0	0.5	1.75	3.5	5.25	7.0
−20 °C		6		6		
−10 °C			6		6	
0 °C	10	6		10		6
+10 °C			6		6	
+20 °C		6		6		

Each replicate contained seven pork chops; one-half of the replicates were fried after irradiation.
